# Altering the redox state of skeletal muscle by glutathione depletion increases the exercise‐activation of PGC‐1*α*

**DOI:** 10.14814/phy2.12224

**Published:** 2014-12-23

**Authors:** Natalie A. Strobel, Aya Matsumoto, Jonathan M. Peake, Susan A. Marsh, Tina‐Tinkara Peternelj, David Briskey, Robert G. Fassett, Jeff S. Coombes, Glenn D. Wadley

**Affiliations:** 1 Exercise and Oxidative Stress Research Group, School of Human Movement Studies, The University of Queensland, St. Lucia, Queensland, Australia; 2 Baker IDI Heart and Diabetes Institute, Melbourne, Victoria, Australia; 3 School of Biomedical Sciences and Institute of Health and Biomedical Innovation, Queensland University of Technology, Brisbane, Queensland, Australia; 4 College of Pharmacy, Washington State University, Spokane, Washington, USA; 5 School of Medicine, The University of Queensland, Brisbane, Queensland, Australia; 6 Department of Physiology, The University of Melbourne, Parkville, Victoria, Australia; 7 Centre for Physical Activity and Nutrition, School of Exercise and Nutrition Sciences, Deakin University, Burwood, Victoria, Australia

**Keywords:** Diethyl maleate, exercise, PGC‐1*α*, reactive oxygen species

## Abstract

We investigated the relationship between markers of mitochondrial biogenesis, cell signaling, and antioxidant enzymes by depleting skeletal muscle glutathione with diethyl maleate (DEM) which resulted in a demonstrable increase in oxidative stress during exercise. Animals were divided into six groups: (1) sedentary control rats; (2) sedentary rats + DEM; (3) exercise control rats euthanized immediately after exercise; (4) exercise rats + DEM; (5) exercise control rats euthanized 4 h after exercise; and (6) exercise rats + DEM euthanized 4 h after exercise. Exercising animals ran on the treadmill at a 10% gradient at 20 m/min for the first 30 min. The speed was then increased every 10 min by 1.6 m/min until exhaustion. There was a reduction in total glutathione in the skeletal muscle of DEM treated animals compared to the control animals (*P* < 0.05). Within the control group, total glutathione was higher in the sedentary group compared to after exercise (*P* < 0.05). DEM treatment also significantly increased oxidative stress, as measured by increased plasma F_2_–isoprostanes (*P* < 0.05). Exercising animals given DEM showed a significantly greater increase in peroxisome proliferator activated receptor *γ* coactivator‐1*α* (PGC–1*α*) mRNA compared to the control animals that were exercised (*P* < 0.05). This study provides novel evidence that by lowering the endogenous antioxidant glutathione in skeletal muscle and inducing oxidative stress through exercise, PGC‐1*α* gene expression was augmented. These findings further highlight the important role of exercise induced oxidative stress in the regulation of mitochondrial biogenesis.

## Abstract (precis)

A number of studies have attempted to elucidate mechanisms for the role of exercise‐induced reactive oxygen species in cell signalling and mitochondrial biogenesis including inhibiting reactive oxygen species production, either by enzymatic inhibitors such as the treatment of allopurinol, or through antioxidant supplementation. Our study is the first to investigate the relationship among mitochondrial biogenesis, cell signalling, and antioxidant enzymes by depleting skeletal muscle glutathione with diethyl maleate (DEM) which resulted in a demonstrable increase in oxidative stress during exercise. The major outcome of our study was that by reducing endogenous antioxidant glutathione content, there was impaired capacity for skeletal muscle to neutralize oxidative stress during exercise, resulting in greater PGC‐1*α* gene expression.

## Introduction

It is well‐documented that exhaustive exercise increases the production of reactive oxygen species (ROS) within skeletal muscle leading to oxidative stress (Bailey et al. 2003, 2004). It has been previously speculated that the increase in exercise‐induced oxidative stress can reduce muscle performance (Bailey et al. 2003). As such, antioxidants have been proposed to reduce exercise‐induced oxidative stress and increase performance (Williams et al. 2006). Recently there have been a number of studies that suggest ROS are important cell signaling molecules, particularly for beneficial exercise‐induced adaptations to skeletal muscle (Gomez‐Cabrera et al. 2005; Irrcher et al. 2009). As a result, the use of antioxidant supplementation may result in a dampening of these positive adaptations initiated by ROS (Gomez‐Cabrera et al. 2008; Paulsen et al. 2014). However, not all research supports this notion (Yfanti et al. 2010; Higashida et al. 2011; Strobel et al. 2011).

Mitochondrial biogenesis (synthesis) is one of the key processes involved in skeletal muscle adaptations to exercise. Peroxisome proliferator activated receptor *γ* coactivator‐1*α* (PGC‐1*α*) is an important coactivator of this process and plays an intrinsic role in mitochondrial biogenesis (Pilegaard et al. 2003; Hood 2009). PGC‐1*α* activates a broad range of both nuclear and mitochondrial encoded genes including nuclear respiratory factor‐1 (NRF‐1), NRF‐2, and mitochondrial transcription factor A (Tfam). Specifically, PGC‐1*α* regulates NRF‐1 and NRF‐2, which in turn regulate Tfam (Joseph et al. 2006). Acute exercise stimulates PGC‐1*α* gene expression, which increases mitochondrial synthesis and adaptations (Baar et al. 2002; Akimoto et al. 2005; Hellsten et al. 2007; Wright et al. 2007). Furthermore, upstream signaling pathways such as phosphorylation of p38 mitogen‐activated protein kinase (p38 MAPK) and cAMP‐response element binding protein (CREB) has been shown to activate PGC‐1*α* (Akimoto et al. 2005; Wu et al. 2006; Irrcher et al. 2008).

A number of studies have attempted to elucidate mechanisms for the role of exercise‐induced ROS in cell signaling and mitochondrial biogenesis. Experimental approaches have included inhibiting ROS production, either by enzymatic inhibitors such as the treatment of allopurinol, or through antioxidant supplementation (Gomez‐Cabrera et al. 2005; Kang et al. 2009; Wadley and McConell 2010; Higashida et al. 2011; Wadley et al. 2013). Some studies suggest that long‐term antioxidant supplementation attenuates markers of mitochondrial biogenesis following endurance training (Gomez‐Cabrera et al. 2008; Ristow et al. 2009; Meier et al. 2013). By contrast, other studies report that short‐term antioxidant supplementation does not influence changes in markers of mitochondrial biogenesis after acute exercise (Hellsten et al. 2007; Wadley and McConell 2010; Higashida et al. 2011; Petersen et al. 2012). Alternatively, allopurinol, a xanthine oxidase inhibitor, has been shown to hamper PGC‐1*α* expression after acute exercise (Gomez‐Cabrera et al. 2005; Kang et al. 2009). However, Wadley et al. recently found that allopurinol did not alter PGC‐1*α* expression after acute exercise and endurance training (Wadley et al. 2013). These data highlight that the role of ROS in skeletal muscle adaptations still remains largely unclear and is further compounded by the variability within exercise protocols, animal versus human models, types and duration of antioxidant supplement and age of subjects (both animal and human).

We adopted an alternative approach to investigate the links between ROS, cell signaling and mitochondrial biogenesis following acute exercise. Specifically, we depleted skeletal muscle antioxidants using diethyl maleate (DEM) to increase oxidative stress during exercise, and measured the resultant changes in markers of mitochondrial biogenesis (PGC‐1*α* and NRF‐2), upstream signaling proteins (p38 MAPK and CREB), and endogenous antioxidants glutathione peroxidase (GPx‐1) and superoxide dismutase 2 (SOD2). We hypothesized that lowering intracellular glutathione would increase ROS production during acute exercise, resulting in an increase in markers of mitochondrial biogenesis, signaling proteins, and antioxidant enzymes.

## Materials and Methods

The University of Queensland Animal Ethics Committee approved this study in accordance with National Health and Medical Research Council guidelines.

### Animals

Ten‐week‐old male Wistar rats (*n* = 46) were purchased from the Central Animal Breeding House (The University of Queensland, Australia). They were housed two per cage for the duration of the study in a 12‐hr light/dark cycle environment. Animals were fed on standard rat chow and tap water ad libitum.

### Experimental protocol

Animals were divided into six groups: (1) sedentary control rats (*n* = 8); (2) sedentary rats treated with DEM (*n* = 8); (3) exercise control rats euthanized immediately after exercise (*n* = 8); (4) exercise rats + DEM (*n* = 8); (5) exercise control rats euthanized 4 h after exercise (*n* = 8); and (6) exercise rats + DEM euthanized 4 h after exercise (*n* = 6). Both DEM and control animals were injected 2 h prior to being sacrificed or exercised (Gerard‐Monnier et al. 1992). DEM rats were given an intraperitoneal injection of 3 mmol/kg body mass which was dissolved in extra light olive oil and control animals were injected with the extra light olive oil (Gerard‐Monnier et al. 1992).

Rats were exercised on a modified treadmill divided into eight lanes separated by clear plastic enclosures. All animals were familiarized for 4 days to treadmill running prior to the start of the study, at a 10% gradient at 16–20 m/min for 30 min. Those rats that were willing to run were placed into the exercise groups. Previous research has shown that this selection process is considered appropriate, because health status and muscle physiology properties do not differ between those rats willing to run or not (Bedford et al. 1979; Lambert et al. 1996). Approximately 72 h after familiarization, the exercising rats ran on the treadmill at a 10% gradient at 20 m/min for the first 30 min. The speed was then increased every 10 min by 1.6 m/min until exhaustion. Exhaustion was defined as the inability of the animal to right itself when it was laid on its side. The time until exhaustion was recorded for each animal (Gomez‐Cabrera et al. 2005; Kang et al. 2009). Animals were weighed and sacrificed with sodium pentobarbital (100 mg/kg i.p.). Under a surgical plane of anesthesia, blood was taken by cardiac puncture, and placed on ice. Samples were centrifuged at 600 × *g* for 10 min, and plasma aliquots were stored at −80°C. We chose to excise red gastrocnemius as it is highly recruited during treadmill running and most mitochondrial adaptations occur in muscles with a high oxidative capacity (Terada and Tabata 2004). Once the muscle was excised it was rapidly frozen in liquid nitrogen and stored at −80°C.

### Preparation of rat tissue

Total RNA was extracted from frozen muscle by use of the Micro‐to‐Midi Total RNA Purification System kit and DNase on‐column digestion (Invitrogen, Carlsbad, CA) as previously described (Strobel et al. 2011). For immunoblotting and mitochondrial enzyme activity, frozen muscle (10:1 buffer/mg muscle) was homogenized as previously described (Wadley and McConell 2007; Strobel et al. 2011) in freshly prepared ice‐cold buffer (50 mmol/L Tris at pH 7.5 containing 1 mmol/L EDTA, 10% vol/vol glycerol, 1% vol/vol Triton X‐100, 50 mmol/L NaF, 5 mmol/L Na_4_P_2_O_7_, 1 mmol/L DTT, 1 mmol/L PMSF, and 5 *μ*L/mL protease inhibitor cocktail [P8340; Sigma, St. Louis, MO]). Tissue lysates were incubated on ice for 20 min and then spun at 16,000 × *g* for 20 min at 4°C. The activities of antioxidant enzymes were measured using frozen muscle samples, which had been homogenized, on ice in 0.1 mol/L sodium phosphate buffer ph 7.0 (10:1 buffer/mg muscle). Samples were spun at 16,000 × *g* for 15 min at 4°C. The supernatant was transferred and analysis was done on freshly prepared homogenates. Total and oxidized glutathione were analysed using frozen muscle tissue which was homogenized in 20 *μ*L of 5% (wt/vol) 5‐sulfosalicylic acid (SSA)/mg tissue and centrifuged at 12,000 × *g* for 5 min at 4°C. The supernatant was diluted 1:5.5 in ddH_2_O. Triethanolamine was added to neutralize the solution to ensure optimal pH for the reaction. Separate aliquots of 100 *μ*L were taken for total (tGSH) and oxidised (GSSG) glutathione measurement. The supernatant sample for the GSSG assay was derivatized in 16 *μ*L of the following solution: 30.8% triethanolamine, 0.4% SSA, and 9% 2‐vinylpyridine in ddH2O (Dudley et al. 2006).

### Plasma F_2_‐Isoprostanes

As a marker of oxidative stress, F_2_‐Isoprostanes were extracted from plasma gas chromatography–tandem mass spectrometry (Briskey et al. 2013). Isoprostanes were extracted from plasma after saponification with methanolic NaOH. Samples were spiked with 8‐iso‐PGF2*α*‐d4 (Cayman Chemicals, Ann Arbor, MI) as an internal standard, and incubated at 42°C for 60 min. Samples were then acidified to pH 3 with hydrochloric acid; hexane was then added and the sample was mixed for 10 min before centrifugation at 3000 × *g*. The supernatant was removed and the remaining solution extracted with ethyl acetate and dried under nitrogen. Samples were reconstituted with acetonitrile, transferred into vials with glass inserts and dried. The samples were then derivatized using 40 *μ*L of a 10% pentafluorobenzylbromide/acetonitrile solution (v/v) and 20 *μ*L of a 10% diisopropylethylamine/acetonitrile solution (v/v), and incubated at room temperature for 30 min. Samples were then dried again under nitrogen before 10 *μ*L of pyridine and 20 *μ*L of a Bis(trimethylsilyl)trifluoroacetamide/Trimethylchlorosilane solution (99:1) (Sigma) were added, and the samples were incubated at 45° for 20 min. Finally, hexane was added and 1 *μ*L of the sample was injected for analysis using gas chromatography mass spectrometry (Varian, Belrose, NSW, Australia) in negative chemical ionization mode. The laboratory intra‐assay coefficient of variation for this assay is 4.5%.

### RT‐PCR analysis

RNA concentration was determined by spectrophotometric analysis. First‐strand cDNA was generated from 0.5 *μ*g RNA using AMV Reverse Transcriptase (Promega, Madison, WI) (Wadley et al. 2001). Following reverse transcription, the remaining RNA was degraded by treatment with RNase H (Invitrogen, Mulgrave, VIC, Australia) for 20 min at 37°C. The amount of single stranded DNA was then determined in each sample and compared against an oligonucleotide standard using OliGreen reagent (Invitrogen), which was incubated in the dark at 80°C for 5 min prior to the measurement of fluorescence (Rhinn et al. 2008b; Strobel et al. 2011). The primer sequences were obtained from gene sequences from GenBank: PGC‐1*α*, AY237127; NRF2*α*, M74515; SOD2, NM_017051.2, and GPx‐1, NM_030826.3 (Table 1).

**Table 1.** Primers for mRNA analyses.

Gene	Forward primer (5ʹ–3ʹ)	Reverse primer (5ʹ–3ʹ)
PGC‐1*α*	ACCCACAGGATCAGAACAAACC	GACAAATGCTCTTTGCTTTATTC
NRF2*α*	CTCGGAGCAGGTGACGAG	TGGACCAGCGTATAGGATCA
SOD2	TGGACAAACCTGAGCCCTAA	GACCCAAAGTCACGCTTGATA
GPx‐1	CGACATCGAACCCGATATAGA	ATGCCTTAGGGGTTGCTAGG

Real‐time PCR using SYBR Green chemistry was performed, using the sequence detector software (Rotor‐Gene v6; Corbett Research, Sydney, NSW, Australia), as previously described (Wadley and McConell 2007). Samples were subjected to a heat dissociation protocol after the final cycle of PCR to ensure that only one product was detected. The mRNA of each gene was normalized to the cDNA content in each sample using the OliGreen assay as described above. This has previously been shown to be a robust and suitable method of normalization that avoids the many problems associated with “housekeeping genes” (Lundby et al. 2005; Rhinn et al. 2008a,b; Strobel et al. 2011).

### Western blot analysis

Lysates were solubilized in Laemmli sample buffer. Equal amounts of total protein, determined by a bicinchoninic acid (BCA) protein assay (Pierce, Rockford, IL) with BSA as the standard, were separated by SDS‐PAGE and electrotransfer of proteins from the gel to PVDF membranes. Blots were probed with anti‐p38 rabbit polyclonal MAPK (Cell Signaling, Hartsfordshire, U.K.), and anti‐phospho‐CREB (Cell Signaling) antibodies. Binding was detected with IRDye 800‐conjugated anti‐rabbit IgG (Rockland, Gilbertsville, PA) or IRDye 680‐conjugated anti‐mouse IgG (Invitrogen, Carlsbad, CA) secondary antibodies. All data are expressed as integrated intensity following infrared detection (Odyssey Imaging system; LI‐COR Biosciences, Lincoln, NE). For p38 MAPK signaling, membranes were then stripped (2% SDS (w/v) in 25 mmol/L glycine, pH 2.0) and reprobed with anti‐phospho‐p38 MAPK rabbit polyclonal antibody (Cell Signaling). As a loading control, blots were then reprobed with anti‐*α*‐tubulin mouse monoclonal antibody, which was not significantly different between groups (Sigma, Castle Hill, NSW, Australia).

### Glutathione and antioxidant enzyme activities

Total and GSSG glutathione concentrations were measured by modifying the method of Dudley (Dudley et al. 2006). Absorbance was recorded on a plate reader (Fluostar Optima, BMG Labtech, Vic., Australia). GPx activity was measured using a modified method for the Cobas Mira spectrophotometric analyser (Roche Diagnostics, Basel, Switzerland) (Wheeler et al. 1990). The method used to measure SOD2 activity was modified from Oyanagu (Oyanagui 1984). Protein concentration was determined using a BCA protein assay (Pierce Rockford, Illinois) with BSA as the standard.

### Statistical analyses

Data were checked for normality using the Shapiro‐Wilk test. If the test was significant, data were log transformed and reanalyzed. Plasma F_2_‐isoprostanes, and skeletal muscle tGSH, ratio of GSSG/tGSH, PGC–1*α* mRNA, NRF‐2 mRNA, phosphorylated p38 MAPK protein, CREB protein, GPx mRNA, GPx activity, SOD2 mRNA, and SOD2 activity were all normally distributed. Skeletal muscle GSSG was normally distributed after log transformation.

An unpaired *t*‐test was used to determine differences in time to fatigue between control exercise and DEM exercise groups. For all other data, a two‐way ANOVA was used to determine an interaction between exercise and DEM treatment and if significant a Tukey's post hoc was completed. If there was no interaction, where applicable, a main effect for exercise or DEM is provided. Results are not reported for all time points. Animals sacrificed directly after exercise were used to determine plasma F_2_‐isoprostanes, and skeletal muscle glutathione and cell signaling proteins (Wadley and McConell 2010). Markers of mitochondrial biogenesis and endogenous antioxidants were measured on animals euthanized 4 h after exercise (Wadley and McConell 2010). The time frame for measuring these markers is similar to other studies and has been shown to be the optimal to determine changes after exercise (Perry et al. 2010; Wadley and McConell 2010). Significance was considered at *P* < 0.05. Normalized data are presented as mean ± SE, and log transformed data as geometric mean ± 95% confidence intervals.

## Results

### Effects of DEM and exercise on markers of skeletal muscle ROS directly after acute exercise

Time to fatigue was significantly decreased as a result of DEM treatment (*P* < 0.05; 68 ± 5 min exercise control [*n* = 16] vs. 51 ± 5 min exercise DEM [*n* = 14]).

**Figure 1.**
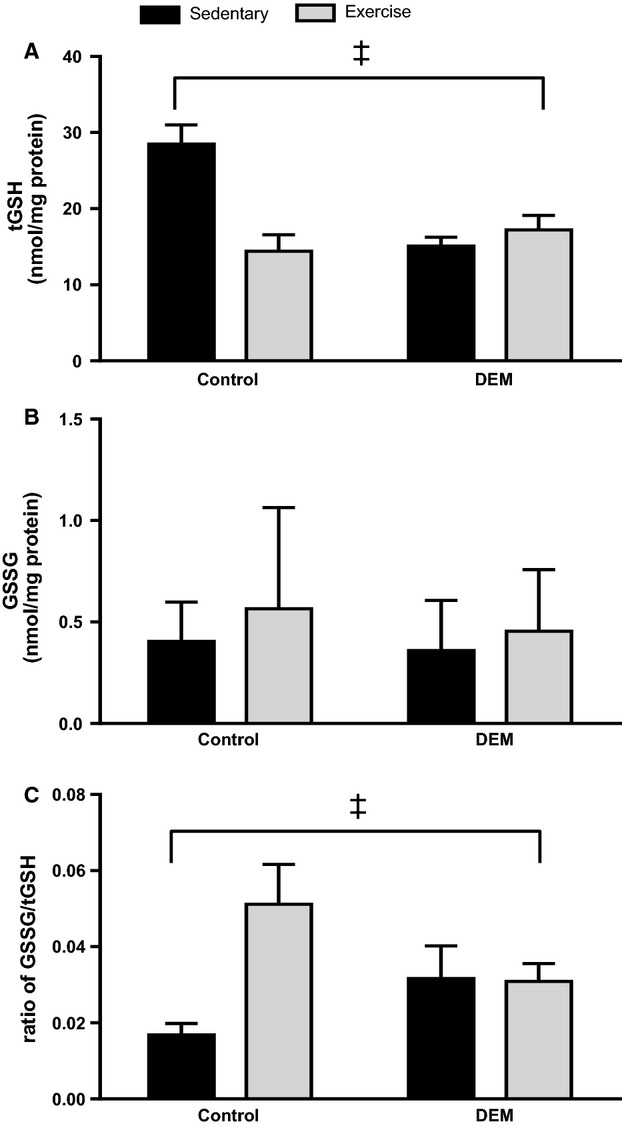
Effects of diethyl maleate (DEM) and exercise on levels of total glutathione (tGSH) (A), oxidised glutathione (GSSG) (B), and the GSSG/tGSH ratio (C). Animals in the post‐exercise groups were sacrificed directly after exercise. Values are mean ± SE for tGSH, GSSG/tGSH ratio and geometric mean (95% CI) for GSSG (*n* = 5–8 for all groups). ^&ddagger;^*P* < 0.05 interaction between exercise and DEM.

**Figure 2.**
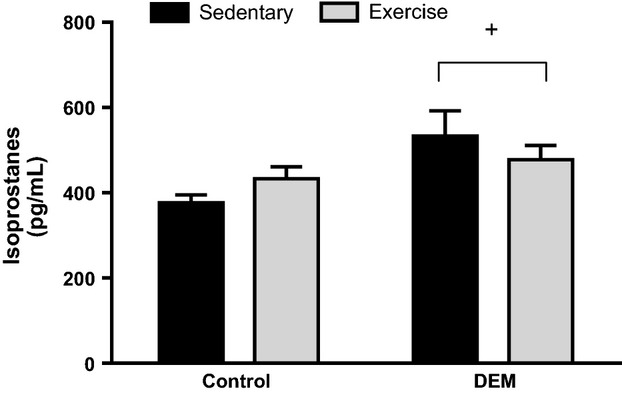
Effects of diethyl maleate (DEM) and exercise on concentration of plasma F_2_‐isoprostanes. Animals in the post‐exercise groups were sacrificed directly after exercise. Values are mean ± SE (*n* = 6–8 for all groups). ^+^*P* < 0.05 main effect for DEM.

For tGSH levels, there was a significant interaction between DEM treatment and exercise (*P* < 0.05; Fig. 1A). In the control (untreated) rats, acute exercise significantly decreased skeletal muscle tGSH levels (*P* < 0.05; Fig. 1A). DEM treatment significantly reduced skeletal muscle tGSH levels compared to untreated rats (*P* < 0.05; Fig. 1A), with exhaustive exercise not reducing these levels any further (Fig. 1A). In the control group, exercise resulted in a greater GSSG/tGSH ratio compared to the sedentary group (*P* < 0.05), yet in the DEM treated animals, GSSG/tGSH ratio did not differ between the exercise and sedentary animals (*P* < 0.05 interaction between exercise and DEM; Fig. 1C). There were no differences in GSSG between groups (*P* > 0.05; Fig. 1B). DEM treatment significantly increased oxidative stress, as measured by changes in plasma F_2_‐isoprostanes (*P* < 0.05 main effect for DEM; Fig. 2).

### Effect of DEM and exercise on mitochondrial biogenesis markers 4 h after acute exercise

**Figure 3.**
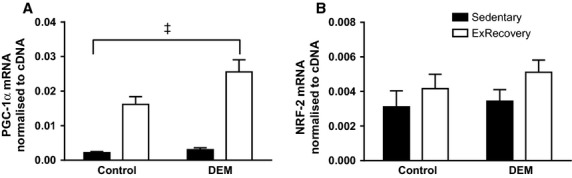
Effects of diethyl maleate (DEM) and exercise on PGC‐1*α* (A) and NRF‐2 (B) gene expression. Animals in the exercise‐recovery groups were sacrificed 4 h after exercise. Values are mean ± SE (*n* = 6–8 for all). ^&ddagger;^*P* < 0.05 interaction between exercise and DEM.

Four hours after exercise, PGC‐1*α* gene expression was significantly increased in both control and DEM treated animals (*P* < 0.05 interaction between exercise and DEM; Fig. 3A). Furthermore, exercising animals treated with DEM showed a significantly greater increase in PGC‐1*α* gene expression compared to the control animals that were exercised (*P* < 0.05 interaction between exercise and DEM). Exercise did not significantly increase NRF‐2 gene expression (*P* = 0.1; Fig. 3B).

### Effect of DEM and exercise on exercise‐induced mitochondrial biogenesis signaling directly after acute exercise

**Figure 4.**
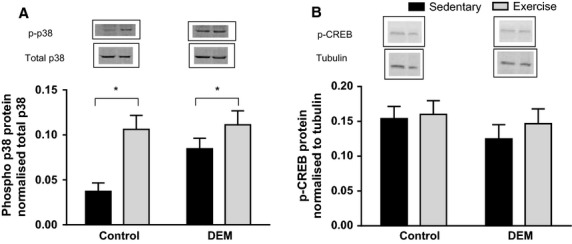
Effects of diethyl maleate (DEM) and exercise on protein content of phosphorylated p38 (A) and phosphorylated CREB (B). Animals in the post‐exercise groups were sacrificed directly after exercise. Values are mean ± SE (*n* = 8 for all groups). Boxes: Western blot showing representative results from 1 rat/group with boxes around blots indicating blots were obtained from different parts of the same membrane. *P < 0.05 main effect for exercise.

Phosphorylation of p38 MAPK was significantly increased after exercise in both the control and DEM treatment groups (*P* < 0.05 main effect for exercise; Fig. 4A). DEM tended to increase the phosphorylation of p38 MAPK (*P* = 0.06; Fig. 4A). Phosphorylated CREB was not altered as a result of exercise or DEM treatment (*P* > 0.05;Fig. 4B).

### Effect of DEM on endogenous antioxidants 4 h after acute exercise

**Figure 5.**
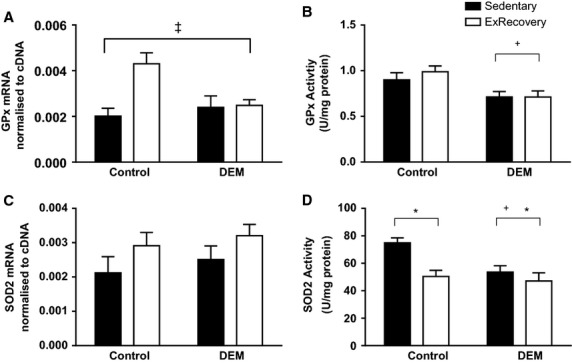
Effects of diethyl maleate (DEM) and exercise on changes in gene expression and enzyme activity of GPx‐1 (A and B) and SOD2 (C and D). Animals in the exercise‐recovery groups were sacrificed 4 h after exercise. Values are mean ± SE (*n* = 6–8 for all groups). ^&ddagger;^*P* < 0.05 interaction between exercise and DEM; *P < 0.05 main effect for exercise; ^+^*P* < 0.05 main effect for DEM.

Diethyl maleate treatment prevented the increase in GPx‐1 mRNA observed in the control (untreated) group following acute exercise (*P* < 0.05 interaction between exercise and DEM; Fig. 5A). In animals treated with DEM, there was a significant reduction in GPx activity levels in the sedentary and exercise groups (*P* < 0.05 main effect for DEM; Fig. 5B).

There was a trend for exercise to increase SOD2 gene expression (*P* = 0.08; Fig. 5C). SOD2 activity was reduced in the control exercise and DEM exercised animals (*P* < 0.05 main effect of exercise; Fig. 5D). In addition, in the DEM groups SOD2 activity was reduced (*P* < 0.05 main effect for DEM; Fig. 5D).

## Discussion

This study is the first to investigate the effects of depleting glutathione and thus increasing oxidative stress on skeletal muscle markers of mitochondrial biogenesis, upstream signaling proteins and endogenous antioxidants following an acute exercise bout. Consistent with our hypothesis, DEM decreased tGSH and GSSG/tGSH. Furthermore, DEM treatment significantly increased oxidative stress during exhaustive exercise as measured by increased plasma F_2_‐isoprostanes. Our main finding was that DEM treatment significantly augmented PGC‐1*α* mRNA following exhaustive exercise. In addition, both DEM and exhaustive exercise significantly lowered GPx‐1 gene expression. DEM also lowered total GPx‐1 and SOD2 activity in both sedentary and exercise groups. DEM treatment also tended to augment the increased phosphorylation of p38 MAPK following exhaustive exercise.

Diethyl maleate treatment decreased skeletal muscle tGSH by approximately 52% which has been previously demonstrated in similar exercise studies using _L_‐butathione‐(S,R)‐sulfoximine (BSO), which also reduces glutathione biosynthesis (Sen et al. 1994; Leeuwenburgh and Ji 1995). The decrease in skeletal muscle tGSH post‐exercise in the control group suggests that exercise alone depleted skeletal muscle glutathione, and is the result of tGSH responding to the oxidative challenge induced by exercise (Lew et al. 1985). Consistent with previous rodent treadmill studies, skeletal muscle GSSG/tGSH ratio was significantly increased in the control animals postexercise, which is indicative of oxidative stress (Wadley and McConell 2010; Wadley et al. 2013). However, the differences in the GSSG/tGSH ratio were predominantly the result of a significant decrease in tGSH. Wadley et al. have previously found that GSSG/tGSH as an indirect marker of ROS production during exercise is more sensitive than other ROS measures, such as protein carbonyls and H_2_O_2_ levels (Wadley and McConell 2010). However, in this instance these markers are not appropriate to determine the increase in ROS in DEM‐treated animals, because DEM reduced both tGSH and GSSG/tGSH. Accordingly, we measured plasma F_2_‐isoprostane concentration as an alternative marker of oxidative stress, which confirmed the expected increase in ROS production within both the sedentary and exercise DEM groups. This finding highlights that DEM treatment induced oxidative stress well above the control (non DEM) supplemented group.

A major finding of this study was that DEM treatment diminished the glutathione pool, resulting in the inability of skeletal muscle to limit oxidative stress, and therefore augmented PGC‐1*α* gene expression following exhaustive exercise. Recent in vitro evidence suggests that increases in PGC–1*α* promoter activity and gene expression are mitigated by increased hydrogen peroxide levels in skeletal muscle (Silveira et al. 2006; St‐Pierre et al. 2006; Irrcher et al. 2009). Given that one of the major roles of glutathione system is to reduce hydrogen peroxide to water, and we diminished the glutathione pool, it is possible that this resulted in an increase in hydrogen peroxide, thus a potential pathway for the changes seen in PGC‐1*α* gene expression. Although there was a trend for exercise to increase NRF‐2 gene expression, DEM treatment did not affect this gene. It is possible that NRF‐2 gene expression is not sensitive to increases in ROS following treatment with DEM.

The signaling proteins, p38 and CREB, are involved in the regulation of PGC‐1*α* gene expression (Wu et al. 2002; Akimoto et al. 2005). Consistent with our previous studies, acute exercise significantly increased p38 MAPK phosphorylation (Wadley and McConell 2010; Wadley et al. 2013). Indeed, an increase in phosphorylation of p38 MAPK, followed by the exercise‐induced increase in PGC‐1*α* gene expression is also similar to that reported by Akimoto et al., providing further support of the functional role of p38 in skeletal muscle adaptations (Akimoto et al. 2005). Skeletal muscle phosphorylation of p38 MAPK also tended (*P* = 0.06) to be higher with DEM‐treatment. It appears that oxidative stress resulting from DEM treatment may have increased p38 MAPK activation. Alternatively, it does not appear that CREB is activated by oxidative stress, which did not change significantly.

In vitro evidence suggests that PGC‐1*α* is a powerful inducer of antioxidants GPx and SOD2. When PGC‐1*α* is gene silenced, there are concurrent decreases in the gene expression of these antioxidants (St‐Pierre et al. 2006). Indeed, as exercise increased PGC‐1*α* gene expression in the controls animals within this study, similar increases were also seen in GPx‐1 and a tendency for increased SOD2 gene expression. These increases have been previously reported in skeletal muscle after acute exercise (Wadley and McConell 2010). DEM‐treatment decreased both GPx‐1 gene expression and GPx activity. Most likely this is the result of the significant reduction in tGSH in the DEM animals. Although we did not expect to see any changes in GPx activity following acute exercise, this measure was used to demonstrate how DEM influences different components of the glutathione system. In the control group, SOD2 activity was reduced after exercise. Several studies have reported no alteration in SOD2 activity after acute exercise (Hollander et al. 2001; Pimenta Ada et al. 2007). Nevertheless, it remains unclear why exercise does not alter SOD2 activity. In addition, DEM decreased SOD2 activity in both the sedentary and exercise groups. We speculate that this overall decrease indicates that SOD2 activity is dependent on glutathione status at rest compared to exercise.

Diethyl maleate significantly decreased time to fatigue by 25%, confirming the findings of others (Kramer et al. 1993; Sen et al. 1994). Both DEM and BSO treatment have previously resulted in reductions in swimming distance (Kramer et al. 1993) and time to fatigue (Sen et al. 1994) respectively. One limitation of the present study was the exercise to exhaustion protocol, where the reduced time to fatigue in the exercise DEM group would have resulted in reduced energy expenditure compared to the exercise controls, and therefore the possibility of reduced exercise‐induced adaptations. Nevertheless, irrespective of the energy expenditure and shorter running time, PGC‐1*α* gene expression following exercise was actually higher in the DEM group. In addition, we also chose to use DEM as a glutathione inhibitor, which also results in the reduction of glutathione in liver, kidney, and brain in addition to skeletal muscle (Gerard‐Monnier et al. 1992). Furthermore, DEM has been shown to reduce glycogen in isolated hepatocytes (Goethals et al. 1983). Given this, evidence recently published suggests that it is possible that the increase in PGC‐1*α* mRNA levels may be attributable to the reduced glycogen, therefore results need to be interpreted with caution (Psilander et al. 2013).

This study has demonstrated novel evidence that by lowering endogenous antioxidant glutathione content, there was impaired capacity for skeletal muscle to neutralize oxidative stress during exercise, resulting in greater PGC‐1*α* gene expression. Therefore, providing in vivo evidence for the important role oxidative stress plays in the regulation of mitochondrial biogenesis.

## Acknowledgments

The authors thank the technical advice and assistance of Mr. Gary Wilson and the School of Human Movement Studies, The University of Queensland for their support.

## Conflicts of Interest

The authors have no conflicts of interest to declare.
